# Probing the influence of graphene oxide sheets size on the performance of label-free electrochemical biosensors

**DOI:** 10.1038/s41598-020-70384-5

**Published:** 2020-08-12

**Authors:** Shimaa Eissa, Jeanne N’diaye, Patrick Brisebois, Ricardo Izquierdo, Ana C. Tavares, Mohamed Siaj

**Affiliations:** 1 Dept. de Chimie et Biochimie, NanoQAM, QCAM/CQMF, Université du Québec à Montréal, Montreal, H3C 3P8 Canada; 2 Institut National de la Recherche Scientifique – Énergie, Matériaux et Télécommunications, 1650, Boul. Lionel-Boulet, Varennes, QC J3X 1S2 Canada; 3 Present Address: Department of Chemistry, Alfaisal University, Al Zahrawi Street, Al Maather, Al Takhassusi Road, Riyadh, 11533 Saudi Arabia

**Keywords:** Materials for devices, Graphene

## Abstract

The integration of graphene materials into electrochemical biosensing platforms has gained significant interest in recent years. Bulk quantities of graphene can be synthesized by oxidation of graphite to graphite oxide and subsequent exfoliation to graphene oxide (GO). However, the size of the resultant GO sheets changes from the parent graphite yielding a polydispersed solution of sizes ranging from a few nanometers to tens of micrometers. Here, we investigate the direct effect of GO sheets sizes on biosensor performance. We separated different GO sheets sizes, and we characterized them via atomic force, scanning electron, Raman and X-ray photoelectron spectroscopies and solid state nuclear magnetic resonance (NMR). As proof of concept, the sensing performance of these GO samples was probed using a well-known ssDNA aptasensor against microcystin-LR toxin and an immunosensor against β-lactoglobulin. The resulting aptasensors and immunosensors are fabricated by using covalent attachment and physical adsorption. We found that the aptasensors fabricated using physical adsorption, the binding signal variation was dramatically increased with increasing the GO sheet size. In contrast, for the aptasensor fabricated using covalent immobilization, the binding signal variation decreased with increasing GO sheet size. However, for the β-lactoglobulin immunosensors, the optimum signals were observed at intermediate GO sheet size. GO sheet size could enhance or inhibit the sensitivity of the graphene-based electrochemical sensors. Our results demonstrate that controlling the size of GO sheets may have a profound impact in specific biosensing applications.

## Introduction

Graphene and related materials have drawn great research interest in recent years because of their exceptional electrical, mechanical and thermal properties^1,2^. Graphene oxide has shown a variety of potential applications in nanoelectronics^3^, protective coatings^4^, polymer composites^5^, catalysis^6^, energy storage devices^7^, drug delivery, optics^8^ as well as sensing and biosensing platforms^9–12^. Several methods have been used for preparation of graphene^13,14^ and the graphene oxide based materials^15,16^. For most applications including biosensors, large graphene quantities with controlled amount of defect, edge and basal planes are required. Oxygen-containing groups at GO edges or surface greatly influence the electrochemical performance of graphene in terms of the heterogeneous electron transfer rate which can be either advantageous or detrimental towards the sensing of a target analyte^17^ and influenced by the percentage of mass incorporations of GO in screen-printed electrodes^18^, and by the amount of C/O moieties dominating the voltammetric response^19^. More importantly, it has been shown that the lateral flake sizes clearly affect the electrochemical activity, and the smaller lateral flake sizes is more electrochemically active. For that, industrial-scale production of graphene is commonly achieved using solution-based approaches^15^. These approaches involve the chemical oxidation of graphite and consequent exfoliation to individual sheets of graphene oxide either via rapid heating or ultrasonication^15^. However, these methods do not usually allow precise control over sheet size and therefore, do not result in monodisperse GO samples^20–22^.

Previous studies have revealed several factors that can influence the size of graphene oxide sheets^23–28^. McAllister et al.^24^ have reported that the size of the GO sheets is not controlled by the size of parent graphite particles used for the synthesis. Dhifaf et al. have shown the effect of the starting material (flakes, ground and powder) on the lateral size of the resulting graphene sheets and their structural characteristics^25^. However, it was shown by Zhang et al.^26^ that the degree of oxidation is a crucial factor that significantly influences the size of the resulting GO particles prepared using Hummers method. Zhao et al.^27^ have also reported a decrease in the GO sheets size with increasing their oxygen content which was explained in terms of higher density of carbon–oxygen bonds that facilitates cracks formation during sonication. Another study has shown that the larger crystal size in the parent graphite yields larger GO sheets with shorter sonication times, regardless of the C/O ratio of the graphite oxide^28^. Moreover, Su et al.^29^ have shown the decrease of graphene oxide sheets size with increasing sonication time. Therefore, because of the numerous variables that may affect the overall synthesis process, the size of GO sheets may vary from synthesis to synthesis and the resultant material is usually polydisperse and contains sizes from a few nanometers to tens of micrometers^21^.

Since the size-controlled synthesis of GO has not been extensively studied so far^19,21,26^, it is essential to understand whether this material has size-dependent properties. Some work has been previously done to explore the size dependent properties of GO flakes. For instance, Kim et al.^30^ have revealed that smaller GO sheets are more hydrophilic due to the presence of more ionized –COOH groups on their edges, and thus the colloidal stability of GO is also size-dependent. It has been also reported that the size of graphene sheets can have significant impact on the behaviour of graphene electrodes and composites^31^. Hicks et al.^31^ have demonstrated that larger graphene sheets have higher conductivity due to the lower contribution of contact resistance^32^. Furthermore, larger sheets shown to be more effective when graphene is used as reinforcement or plate like fillers in composites^33,34^. Pumera et al.^35^ have also recently studied the effect of parent graphite particle size on the electrochemistry of thermally reduced graphene oxide (TR-GO). It was demonstrated in this report that the size of parent graphite particles has no clear effect on the defect density, amount of oxygen containing groups and rate of heterogeneous electron transfer (HET) of ferro/ferricyanide redox probe. Instead, it was shown that the electrochemical behaviour depended on the structural properties of the produced graphene materials irrespective of their sheet size^35^. Despite the relatively large number of graphene-based electrochemical biosensors reported to date, no systematic study has yet examined the extent to which varying graphene oxide sheet size impacts the biosensor performance.

Label-free electrochemical biosensors are devices that monitor the changes in electrical properties of the surface when a target bioanalyte interacts with a probe-functionalized surface without any labelling. Unlike the labelled biosensors that require extra time, expense, and sample handling, label-free biosensors are simpler, easier, lower cost and can enable detection of target-probe binding in real time^36^. Hence, significant graphene-based label-free biosensors are being developed which motivates us to focus on such systems^9,37^.

In this paper, we investigate in detail the effect of varying the lateral size of graphene oxide sheet produced from graphite route on the performance of two representative label-free electrochemical aptasensing and immunosensing systems employing either physical or covalent immobilization. The work is a proof of concept to show the direct effect of the GO sheets sizes on both sensors behavior. First, we separated GO bulk solution prepared using modified Hummers method^38^ in various graphene oxide fractions with different size ranges. The structural properties of the separated sheets were characterized using solid state NMR, Raman spectroscopy and X-ray photoelectron spectroscopy. Then, the separated GO fractions were deposited on the surface of screen-printed electrodes and used for the biosensors fabrication.

## Results and discussion

Since the production of monodisperse GO remains a major challenge, a post-processing approach has been performed herein after GO synthesis for the flake’s separation. To avoid introducing additional impurities to the highly adsorbing GO sheets, we did not use a density gradient ultracentrifugation for the separation step^39^. Moreover, the gradient making medium that is often used for this method such as sucrose^39^ can result in the reduction of GO.

To prepare size-selected GO suspensions, we used two-step separation methodology. First, an ultracentrifugation step was carried out, which takes advantage of the difference in sedimentation rate between various sized flakes. When a GO suspension in water is placed on a centrifuge tube, the action of centrifugal force results in faster sedimentation rate of the larger and heavier sheets, while slowing down the smaller sheets. Thus, the separation of GO sheets with different lateral sizes can be obtained by collecting two fractions along the centrifuge tube. This process is repeated for each collected fraction and again separated. An image of a tube after centrifugation is shown in Figure S1a. However, based on SEM characterisation of some separated fractions (Figure S1b), we noticed that the centrifugation step alone was not enough to achieve efficient GO’s separation over a specific range of sizes. For that a second step was needed to ensure effective separation, thus, the GO fractions were filtered by using membranes with different pore sizes.

**Figure 1:**
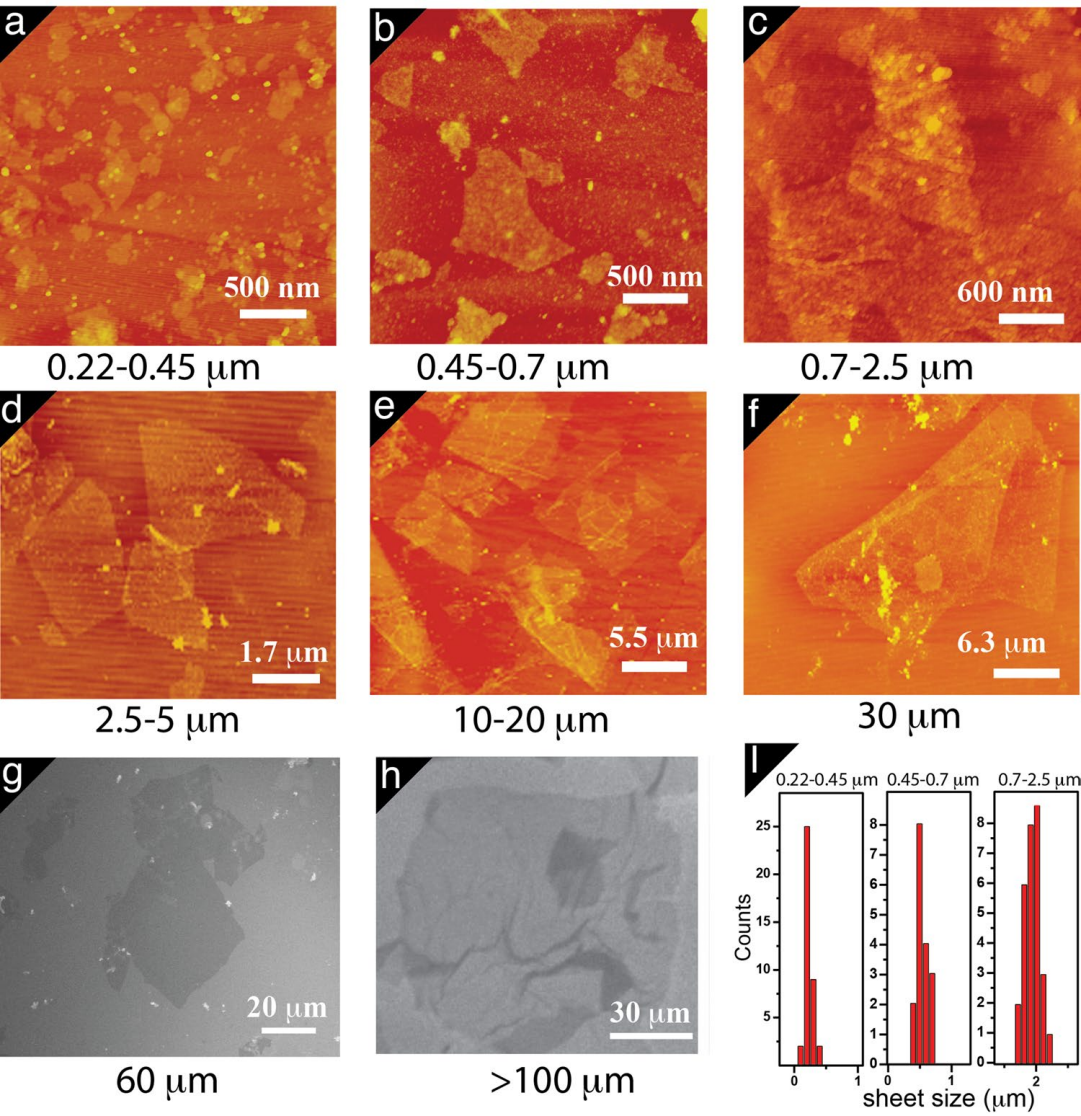
A–H ScanAsyst-mode AFM and SEM images of different GO fractions and (I) Size distribution profile of three representative samples.

The separated GO sheets were then characterized using atomic force microscopy (AFM) and scanning electron microscopy (SEM) to determine the sheets size and thickness and to evaluate the surface morphology. AFM and SEM images of the GO sheets on cleaved mica surfaces showed that the separated GO flakes were diverse and irregular in shape (Fig. 1). The lateral dimensions of the GO flakes determined using AFM and SEM images confirmed the successful separation of different GO samples with specific size ranges. The size distribution of the GO sheets obtained from statistical analysis of the AFM and SEM images (examples are shown in Fig. 1I) indicates that the sheet sizes maximum are mostly 0.22, 0.45, 0.7, 2.5, 5, 10, 30, 60, and > 100 μm. Cross-sectional profiles from the AFM images (Fig. 1d) reveal that all the GO sheets have height ranging from 0.7 to 1.1 nm, which is characteristic of a fully exfoliated GO sheet^26,38^. This indicates that most of the separated GO sheets were single layers. SEM images for three different GO-modified electrodes prepared using different sized sheets (0.22, 0.7, > 100) indicates different surface morphology (Figure S2).

Other structural properties of the GO sheets such as defect density and oxygen functionalities which may affect their electrochemical properties were examined using Raman spectroscopic measurements, XPS and solid state ^13^C NMR, respectively. As shown in Figure S3, the Raman spectra of all GO materials exhibited a G-band at 1,580 cm^−1^ and a D band at 1,350 cm^−1^ associated with the sp^2^-hybridised carbon vibrations and sp^3^-like defects in the backbone, respectively. The degree of disorder in the carbon structure is usually estimated by the ratio between the D and G band intensities (D/G ratio). The results showed that the D/G ratio of most of the GO samples were close in value. The samples with the smallest lateral sizes (0.22 and 0.45 μm) exhibited the highest D/G ratios (0.81) which indicates the presence of more defects presumably due to the presence of smaller sp^2^ domains on the smaller GO sheets. With increasing the GO sheet size, the D/G ratio was slightly decreased to 0.79 and 0.77 for the 0.7 and 2.5 μm GO samples, respectively. Then a ratio of 0.75 was obtained for all the larger sheets (> 5 μm) suggesting similarity in the average amount of defects for all the large GO samples.

**Figure 2:**
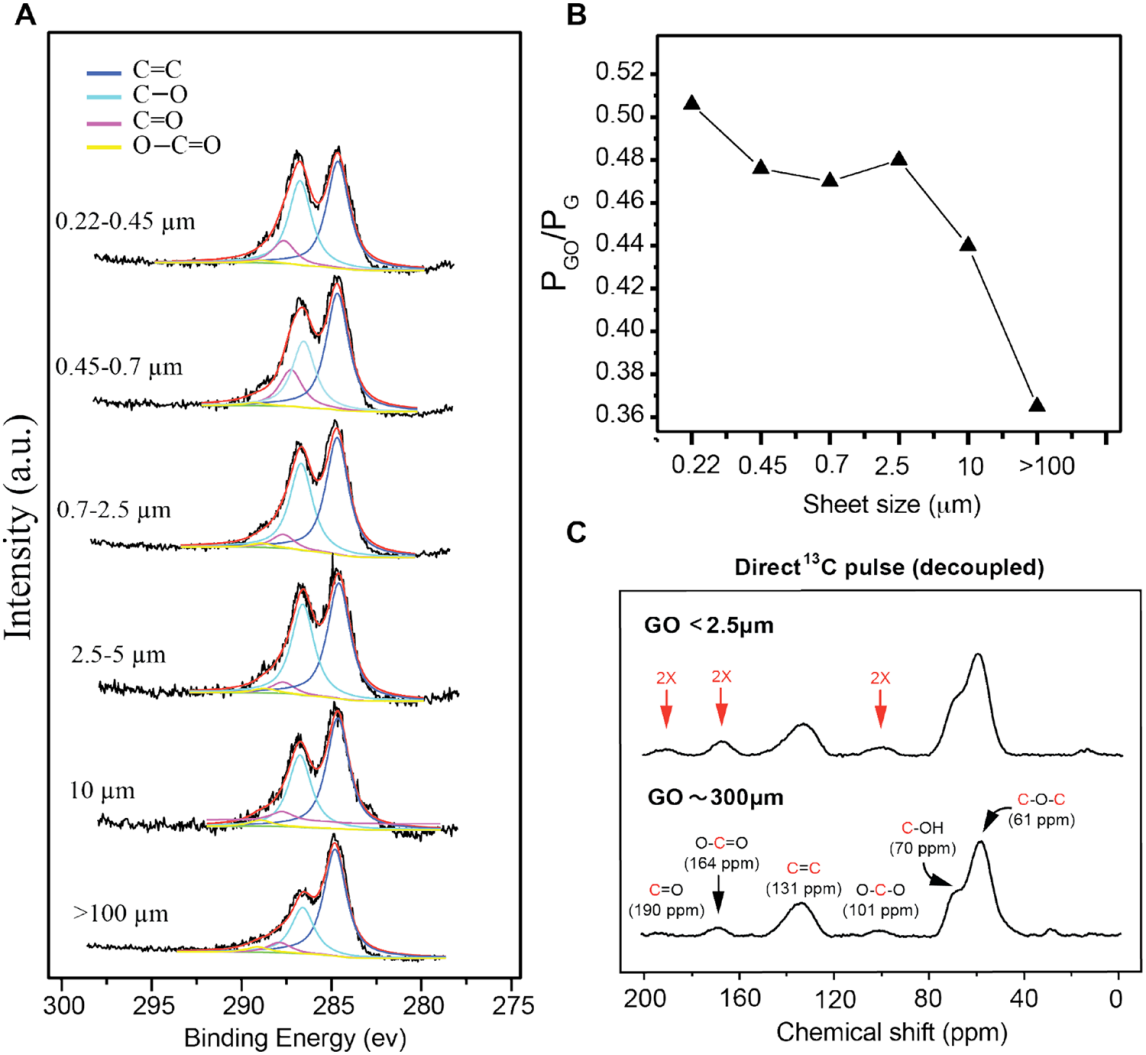
(**A**) XPS C 1s spectra of different sized graphene oxide sheets, (**B**) a plot of P_GO_/P_G_ versus the size of GO sheet and (**C**) ^13^C NMR spectra for the smallest (0.22 μm) and largest (> 100 μm) GO sheets.

XPS was then used to characterise the oxygen functionalities of the GO samples. We calculated the C/O ratio (Figure S4) to estimate the relative degree of oxidation of the different samples. However, these results are not very reliable because it is hard to fully dehydrate GO^36^. Therefore, the ratios of the total peak area of all oxidized carbon divided by total C1s spectral area (P_GO_/P_G_) were compared for all samples^41^. As shown in Fig. 2A, each C1s spectrum was deconvoluted into four peaks at binding energies of 284.5, 286.5, 288.0 and 289.0 eV corresponds to sp^2^ C=C, hydroxyl/epoxide C–O, carbonyl C=O, carboxylic O–C=O, respectively. It can be seen that the peaks for the various carbon–oxygen functional groups diminish from smaller sized GO samples (0.22 μm) to the larger sized sheets (> 100 μm). A plot of the P_GO_/P_G_ ratio versus GO sheet size (Fig. 2B) shows a gradual decrease in the degree of oxidation with increasing sheet size.

In order to confirm these results, we performed high-resolution SSNMR ^13^C analysis for the smallest (0.22 μm) and the largest (> 100 μm) sized GO sheets. Figure 2C shows SSNMR ^13^C spectra of two samples. Three major peaks located at 61, 70, 131 ppm were observed in the SS NMR spectra and assigned to epoxide ^13^C, ^13^C–OH and sp^2 13^C, respectively as previously reported by Brisebois et al.^38^ Well-resolved minor peaks were also observed at 101, 164, and 190 ppm, which are attributed to the O–C–O, O–C=O and C=O, respectively^42^. A thorough analysis for Fig. 2B shows clearly that the degree of oxidation for the smallest sized sheets sample is higher than the largest GO sheets. Particularly, for the smallest sized sheets, the integration of the peaks at 101, 164, and 190 ppm is almost double than those observed in the largest sized sheets. This data confirms that samples with the smaller sheets have more carboxylic groups than those with larger sheets.

**Figure 3:**
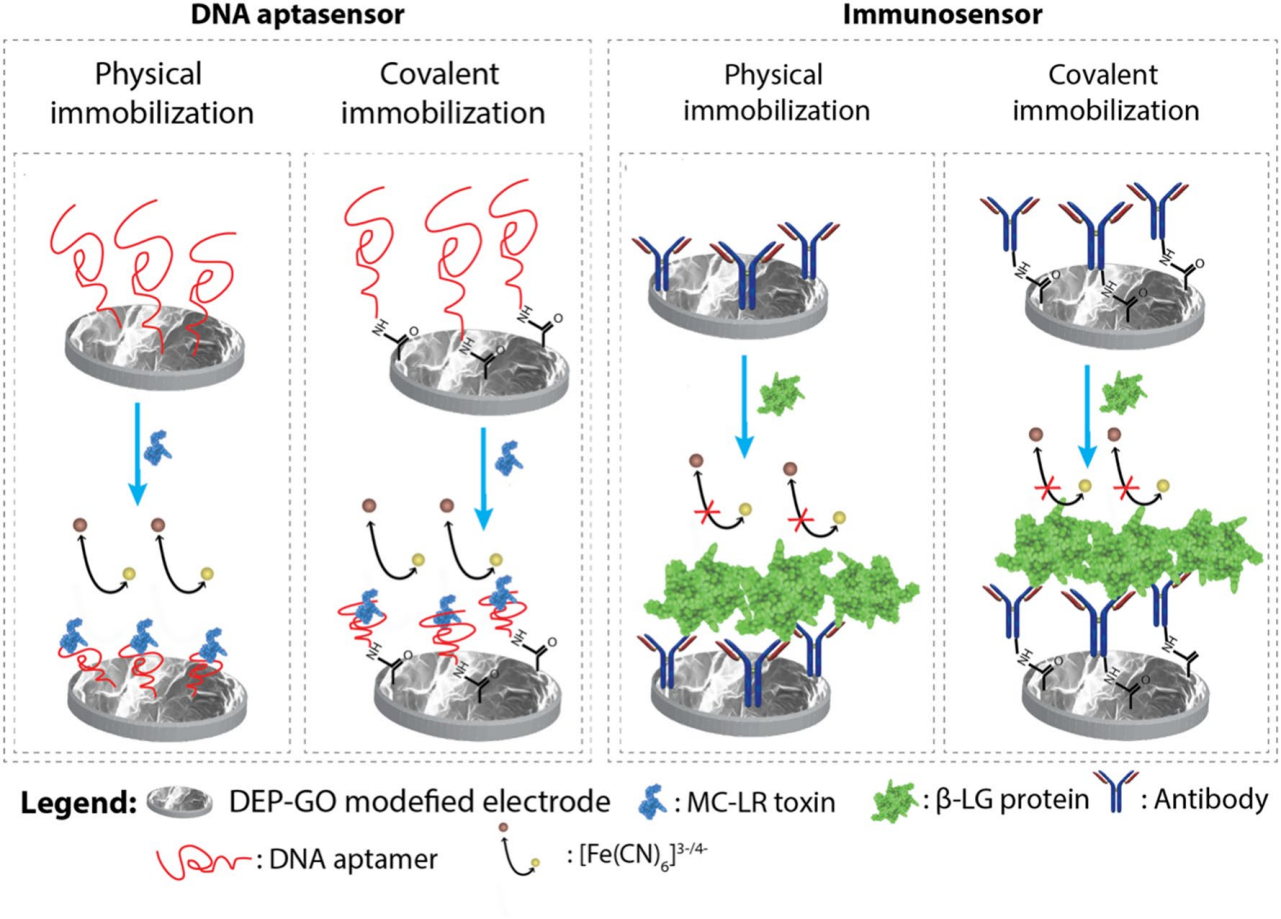
Representation of the MC-LR aptasensors and β-LG immunosensors.

After GO samples’ characterization, we proceeded to systematically investigate their electrochemical biosensing performance. For that, we studied two representative label-free biosensors –an immunosensor directed against the milk protein β-LG and a DNA aptasensor directed against the small molecule MC-LR toxin. The immuonsensor is comprised of a polyclonal antibody against β-LG and the aptasensor involves a 60 nucleotides DNA aptamer sequence that binds specifically to MC-LR showing a K_d_ of 50 nM^43^. The two platforms were fabricated as shown in Fig. 3 by attaching the aptamer or the antibody onto the GO-disposable electrochemical printed (GO-DEP) electrodes employing either physical adsorption (Apt/Phys, Imm/Phys) or covalent attachment immobilization protocol (Apt/Cov, Imm/Cov).

**Figure 4:**
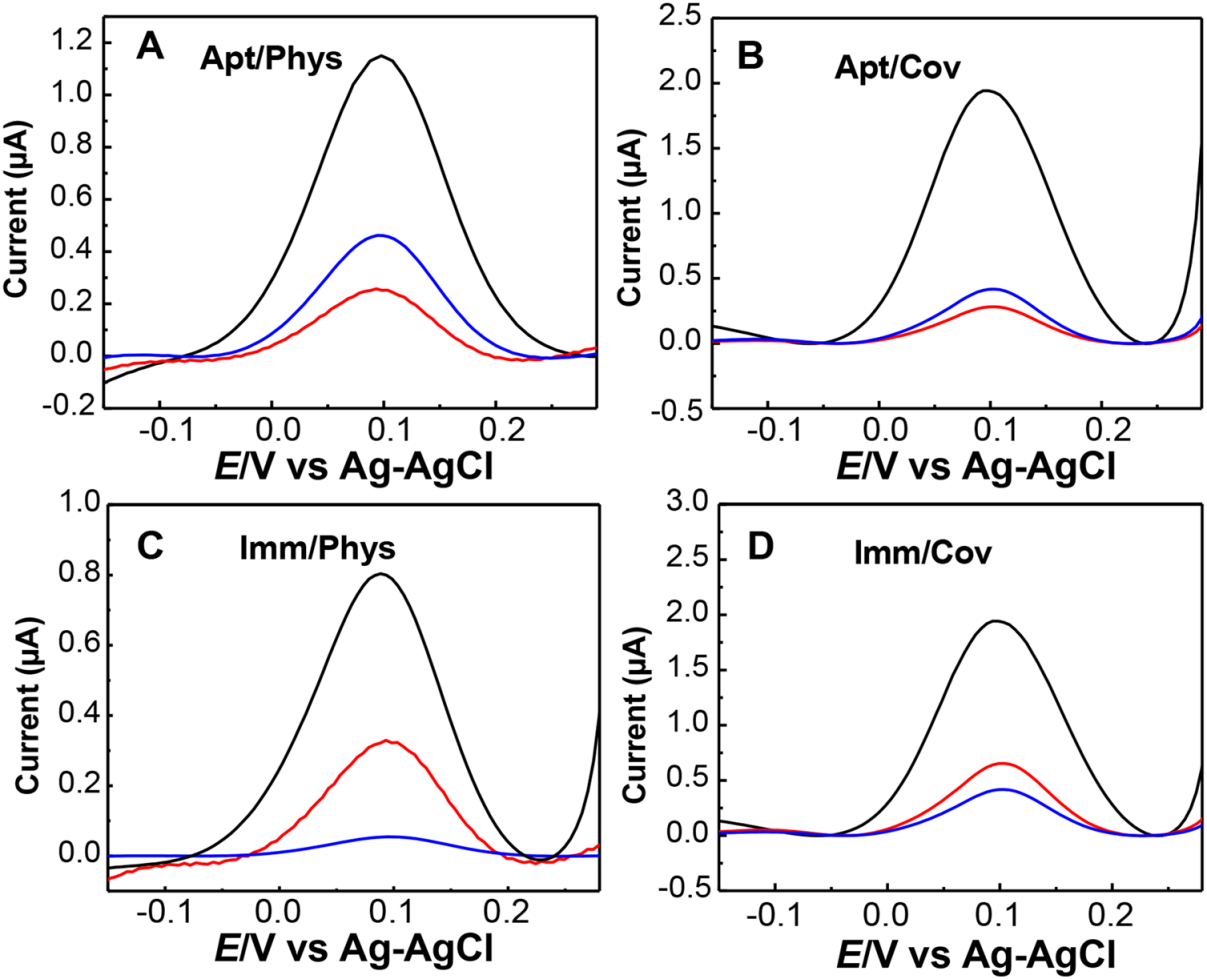
SWVs of the aptasensor fabricated using physical adsorption (**A**) and covalent immobilization (**B**) for bare GO-DEP electrodes (black), MC-LR aptamer-functionalized electrodes (red), and after MC-LR toxin incubation (blue) (concentration of the aptamer, 10 μM; concentration of MC-LR, 1 nM); SWVs of the immunosensors fabricated using physical adsorption (**C**) and covalent immobilization (**D**) for bare GO-DEP electrodes (black), β-LG antibody-functionalized electrodes (red), and after β-LG incubation (blue) (concentration of the antibody, 10 μg/ml; concentration of β-LG, 1 μg/ml). All measurements were done in 1 mM [Fe(CN)_6_]^4−/3−^ probe in PBS, pH 7.4.

We employed here square wave voltammetry for the electrochemical measurements in the presence of the [Fe(CN)_6_]^3−/4−^ redox couple. The square wave voltammetry (SWV) characterization of the different experimental steps for the biosensors are shown in Fig. 4. Figure 4A shows the SWV of the aptasensor fabricated using physical adsorption (Apt/Phys) via the π–π stacking interactions between the graphene hexagonal cells and the nucleobases of the DNA. Figure 4B shows the SWV of the aptasensor fabricated using covalent attachment of the amine-modified microcystin aptamer (NH_2_-MCAP) to the carboxylic groups on the graphene surface via 1-ethyl-3-(3-dimethylaminopropyl) carbodiimide hydrochloride (EDC)/ N-hydroxysuccinimide (EDC/NHS) chemistry (Apt/Cov). The SWV of [Fe(CN)_6_]^3−/4−^ reduction on the bare GO-DEP electrodes is characterized by a well-defined cathodic peak (black lines). The intensity of the reduction peak was decreased upon the immobilization of the aptamer on the GO electrodes due to the shielding of the GO surface as well as by the electrostatic repulsion between the negatively charged phosphate backbone of the DNA aptamer and the [Fe(CN)_6_]^3−/4−^ anions (Fig. 4A,B, red lines). However, upon MC-LR binding, the [Fe(CN)_6_]^3−/4−^ reduction peak current increased (Fig. 4A,B, blue lines). This indicates an enhancement in electron transfer efficiency between the [Fe(CN)_6_]^3−/4−^ redox probe and the GO electrode which is presumably attributed to a change in the aptamer conformation induced by the target as previously reported^41^.

For the immunosensors (Imm/Phys, Imm/Cov) as shown in Fig. 4C,D, a decrease in the [Fe(CN)_6_]^3−/4−^ reduction peak was observed after the β-LG antibody immobilization on the GO electrode due to the blocking effect of this bulky protein (red lines). Upon β-LG binding, a further decrease in the peak current was observed (blue lines) as a result of the additional steric hindrance induced by the bound β-LG molecules. Moreover, the negative charge of the β-LG molecules at pH 7.4^44^ acts as an electrostatic barrier for the electron transfer which also contributes to the decrease in the current. Thus, the change in the electron transfer efficiency of the [Fe(CN)_6_]^3−/4−^ redox probe upon target binding which results in either increase (aptasensors) or decrease (immunosensors) in the reduction peak current represent the basis of the electrochemical sensing.

**Figure 5:**
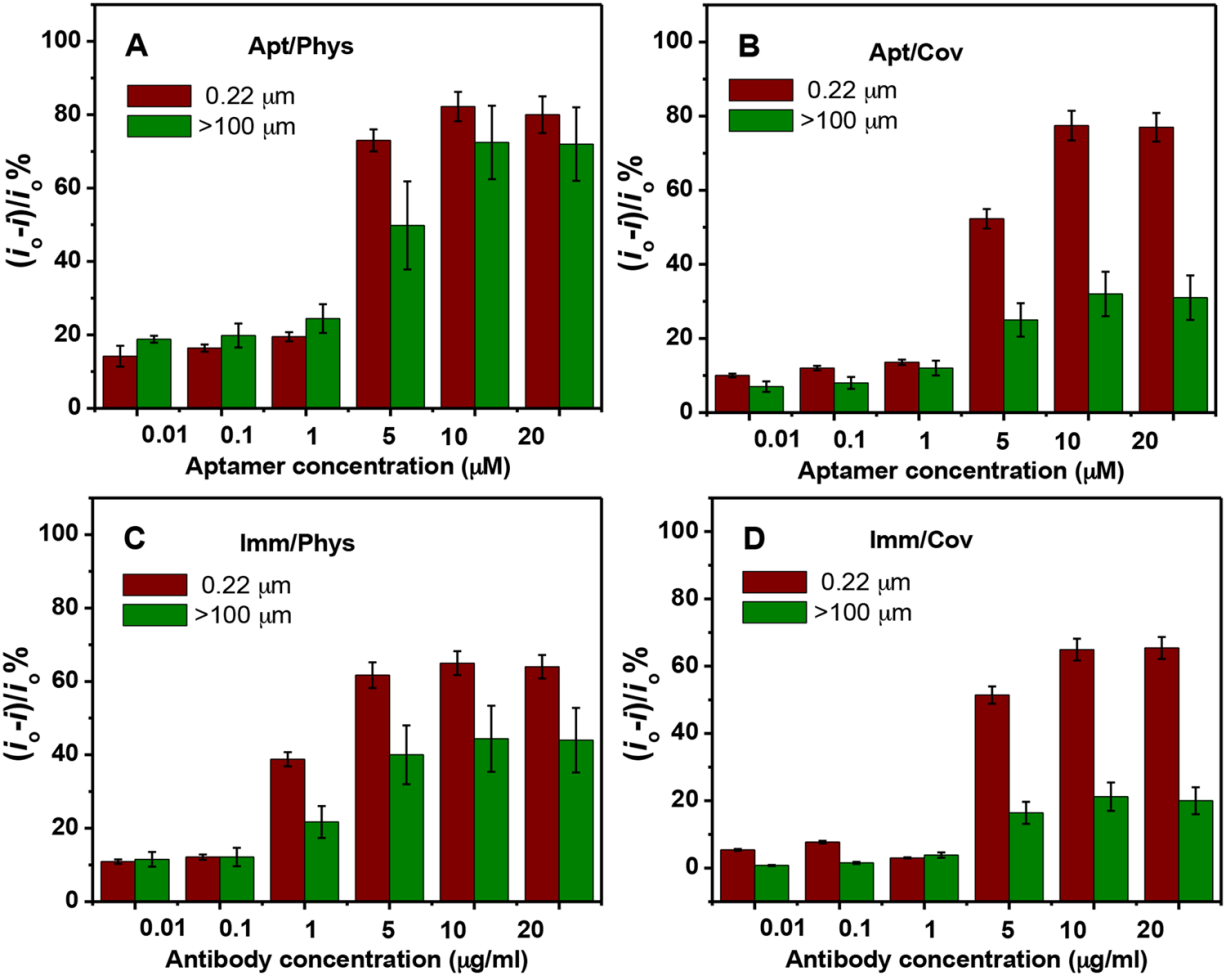
Comparison of the change in the SWV peak current towards the various amounts of (**A**) MCAP, (**B**) NH_2_-MCAP, (**C**) β-LG antibody and (**D**) covalently immobilized β-LG antibody deposited onto graphene oxide modified DEP electrode surface. Signal is represented as (*i*_o_ − *i*)/*i*_o_%. Red columns represent the smallest GO sized sheets and green columns represent the largest GO sized sheets. Error bars correspond to duplicate measurements. All measurements were performed with 1 mM [Fe(CN)_6_]^4−/3−^ in PBS buffer, pH 7.4 at room temperature.

First, MC aptamer and β-LG antibody concentrations were optimized in each case to establish the optimum amount to be used for the aptasensors and immunosensors fabrication. As shown in Fig. 5A–D, SWV peak current variations ((*i*_o_-*i*) /*i*_o_%, where *i*_o_ is the peak current of the bare GO electrodes and *i* is the peak current after bioreceptor immobilization) obtained from the experiments at different aptamer and antibody concentrations were analyzed and depicted in the form of histograms for the four cases. The experiments were performed using both small and large GO sheets (0.22 and > 100 μm) for comparison. In all cases, we observed an increase in the current variation with increasing the amount of aptamer or antibody used for immobilization due to the increased shielding of the GO surface by the probes. It can be concluded that the optimum amount of aptamer or antibody to be immobilized onto the GO electrodes to ensure maximum surface coverage are 10 μM and 10 μg/ml, respectively. Despite that a similar trend was seen for all cases, a higher current variation was obtained for the aptasensors. This is likely due to the difference in the electric charge between the aptamer and antibody. While the negative charge of the aptamer causes an electrostatic repulsion with the redox probe enhancing the decrease in the current, the positive charge of the antibody may attract the redox probe causing less signal change. Besides, the influence of the change in the GO sheet size on the signal was more pronounced on the biosensors prepared by covalent immobilization than on the physical adsorption biosensors. We believe that such difference is induced by the difference in the amount of carboxylic groups that are used for the covalent immobilization of the probe. Therefore, the smaller sized GO electrodes have shown more signal variation because of the presence of more edges on their surfaces that contains higher number of carboxylic groups, as confirmed by both the XPS and SS NMR data above, which in turn leads to an increase in the number of immobilized probes.

The figure of merit of the developed biosensors is the signal gain or suppression observed at a given target concentration. For better comparison of the experiments, the signal is expressed as the relative increase (aptasensor, (*i*_target_ − *i*_aptamer_)/*i*_aptamer_% ((*i*_p_ − *i*)/*i*%)) or decrease (immunosensor, (*i*_Ab_ − *i*_target_)/*i*_Ab_% ((*i* − *i*_p_)/*i*%)) in peak current upon addition of the target from the original signal observed in the absence of the target. We thus now focus on the effect of varying GO sheet size on this measure for the four studied biosensing cases at the optimized probes concentrations.

In general, improved biosensor response signal can be obtained through changes in the immobilized bioreceptor binding efficiency or through changes in the electron transfer efficiency of the redox probe to the GO surface. For example, the signal gain of the MC-LR aptasensors will increase if electrons transfer from the redox probe to the GO surface after target binding is enhanced or if the fraction of the aptamers/target complex increased. However, an increase of the signal suppression of the β-LG immunosensor will occur if the electron transfer was more retarded after analyte binding or if the fraction of bound antibody on the GO surface increased.

**Figure 6:**
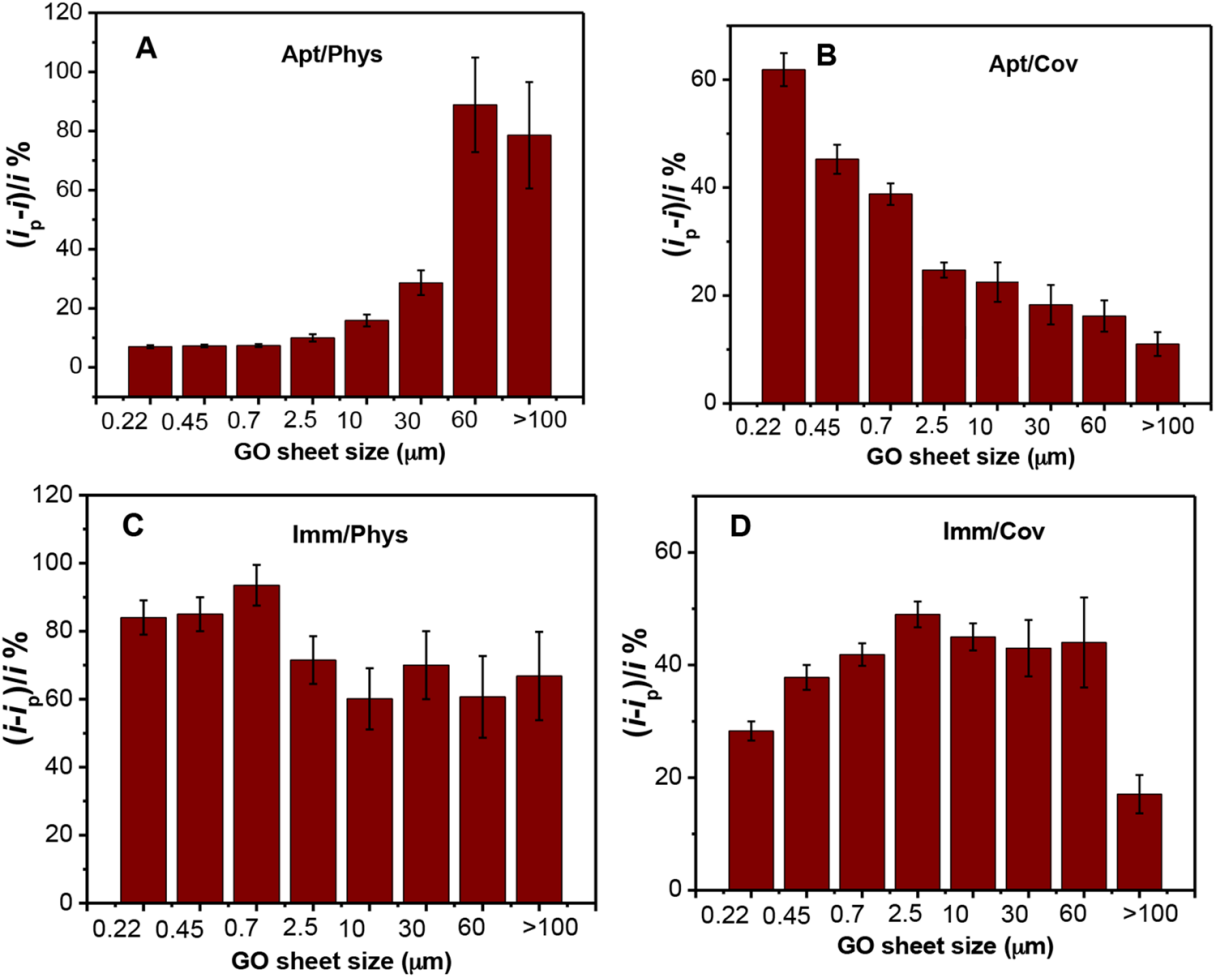
Comparison of change in SWV peak current of the different GO sized-sheets electrodes after the incubation with MC-LR (1 nM) or β-LG (1 μg/ml) for the four different cases. The concentrations used to prepare the sensors were 10 μM for the aptamer and 10 μg/ml for the antibody. Binding signal response represented as (*i*_p_-*i*)/*i*% for the aptasensors fabricated by physical adsorption (**A**), and covalent immobilization (**B**) and as (*i* − *i*_p_)/*i*% for the immunosensors fabricated by physical adsorption (**C**), and covalent immobilization (**D**). The error bars represent the standard deviation of 3 measurements.

It can be clearly seen from the histograms, Fig. 6, that the biosensors response signal is a strong function of the GO sheet size. As shown in Fig. 6A (Apt/Phys), the smallest size GO gives the lowest signal. With increasing the size of the GO sheet, we observed a gradual increase in the aptasensor response. However, the opposite trend is observed for the Apt/Cov (Fig. 6B) where the smallest sized-GO sheets show the highest signals and the signal decreases with increasing the sheet size with almost comparable signals obtained for the larger sizes (> 2.5 μm). This signal enhancement in the Apt/Phys case at larger GO sheet size seems to be associated with the increased efficiency of the electron transfer on the larger sheets that showed less degree of oxidation as confirmed by the XPS and NMR results. The enhancement in electrons transfer efficiency of the [Fe(CN)_6_] ^4−/3−^ redox couple with decreasing the oxygen content on the graphene electrodes has been reported previously^45^. This is attributed to the less electrostatic repulsion between less oxidized graphene and the negatively charged [Fe(CN)_6_]^4−/3−^.

In contrast, for the Apt/Cov case, the covalent immobilization of the aptamers on the graphene via the carboxylic groups located on the graphene edges leads to a higher amount of immobilized probes on the small-sized GO platforms (Fig. 5B) and therefore, more binding signal (Fig. 6B). The crowding of the aptamer on the small-sized GO surface may lead to the hindrance of target accessibility^46^, an effect that might be expected to reduce the sensor gain. However, this steric hindrance effect was not pronounced in this case presumably due to the small size of MC-LR molecule, in addition to the folding of the aptamer occurring upon binding.

However, for the Imm/Phys, as shown in Fig. 6C, the optimal signaling (93%) is obtained at intermediate GO sheet size (0.7 μm), with signaling decreasing at both smaller and larger sheet sizes. For the Imm/Cov, as shown in Fig. 6D, optimal signaling (50%) is also obtained at intermediate GO sheet size (2.5 μm). This may arise from two competing factors: while smaller sized GO sheets allow the immobilization of more antibodies (Fig. 5C,D), which in turn may lead to a reduction in the affinity of some antibodies due to crowding, larger-sized GO may reduce the signal suppression due to the higher electron transfer efficiency of [Fe(CN)_6_]^4−/3−^ on the large sheets. These results indicate that proper match between the GO sheet size, the immobilization protocol of the probe, and target size has to be taken into consideration when designing a biosensor in order to obtain the best performance.

**Figure 7:**
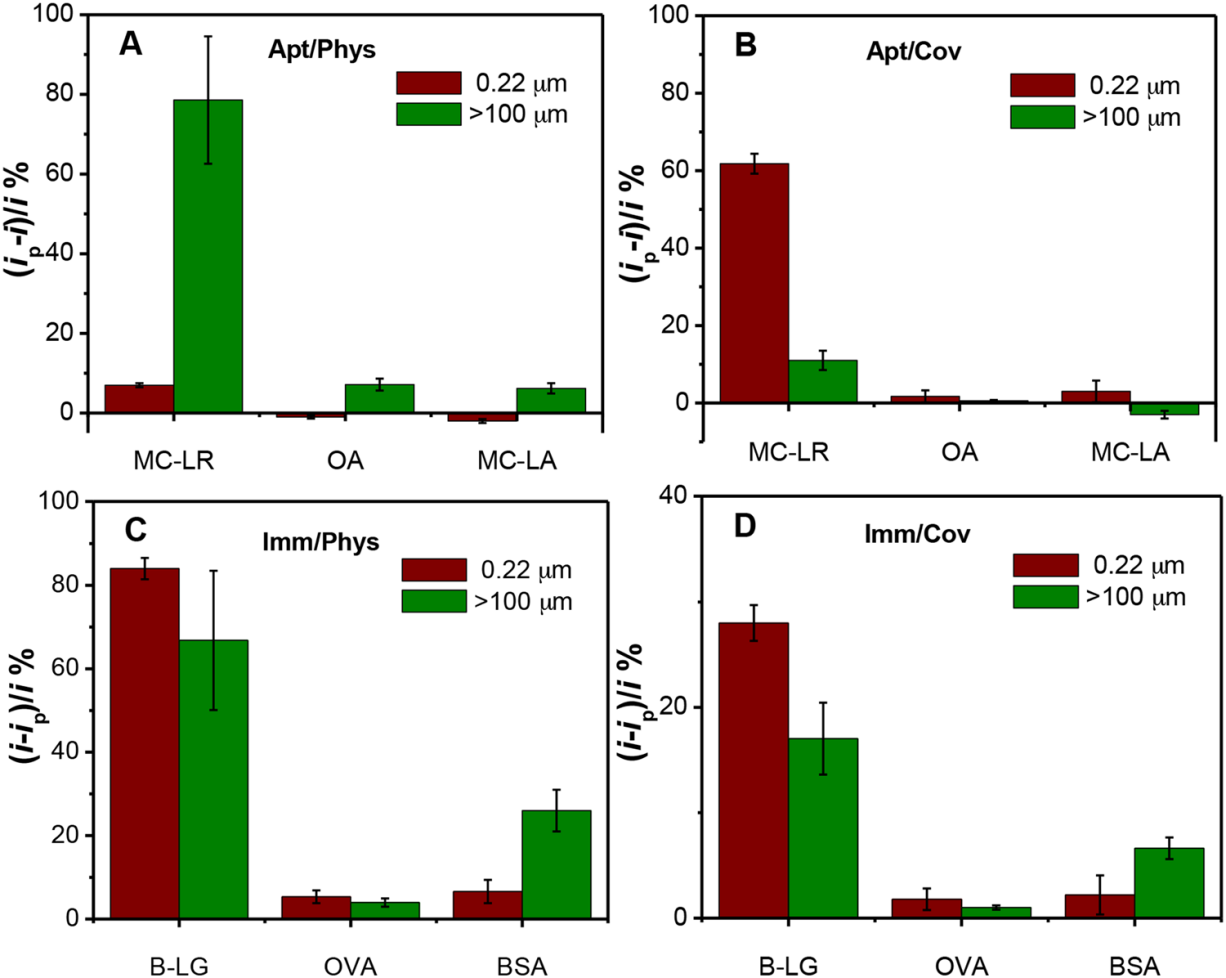
Selectivity experiments of the biosensors prepared using smallest GO sized sheets (red columns) and largest GO sized sheets (green columns); response of the aptasensors fabricated using physical adsorption (**A**) and covalent immobilization (**B**) to 1.0 nM of MC-LR, OA, and MC-LA; response of the immunosensors fabricated using physical adsorption (**C**) and covalent immobilization (**D**) to 1.0 μg/ml of β-LG, OVA, and BSA. The concentrations used to prepare the sensors were 10 μM for the aptamer and 10 μg/ml for the antibody. The error bars represent the standard deviation of 3 measurements.

The extent to which GO sheet size changes the response signal of the studied biosensors raises the parallel question whether the selectivity of biosensors is also sensitive to this parameter. To answer this question, the selectivity of the four biosensors was studied at the smallest (0.22 μm) and the largest (> 100 μm) GO sheet sizes. The MC aptasensors (Apt/Phys and Apt/Cov) were incubated with OA and MC-LA as non-specific toxins, which have similar molecular weight. The β-LG immunosensors (Imm/Phys and Imm/Cov) were incubated with OVA and BSA as non-specific proteins. Figure 7 shows a comparison of the relative change in the peak current for the specific and non-specific targets at the two GO sheet sizes for the four studied cases. For all cases, we observed higher response for the specific analytes against the non-specific analytes. Higher difference between the signal of the specific analytes and the non-specific analytes was obtained with the small-sized GO sheets than the large sheets, particularly for the immunosensors cases. The results indicate good selectivity for the aptasensors prepared by either physical adsorption or covalent immobilization. Another study by Pumera et al.^47^ has shown comparable results of the physical and covalent immobilization of an aptasensor against thrombin unlike a poor response observed by affinity immobilization. However, the immunosensors fabricated on the larger sized GO sheets does not display satisfactory selectivity towards β-LG compared to the smaller sheets likely due to the non-specific adsorption of the protein on the large sheets. Selectivity measurements were also done using the optimized GO sheet size for all four cases and the results showed good selectivity of the assays (data not shown).

The standard deviations of the signals for the four platforms using the small sized GO sheets were ranging from 5.0 to 7.0% (n = 3) indicating good reproducibility of the sensors. However, the standard deviations of the signals using the large-sized GO sheets were ranging from 15 to 25% (n = 3), suggesting poor reproducibility of these sensors. We attribute this low reproducibility to the inhomogeneity of the large-sized GO sheet samples. Despite producing the highest signal in the Apt/Phys case, the larger GO sheets tend to aggregate due to the low hydrophilicity of these sheets, which in turn leads to inhomogeneous dispersion.

In order to have better idea on the effect of GO sheet size on the sensitivity of the sensors, we have also studied the responses of the sensors at different concentrations of the analytes. The aptasensors and the immunosensors prepared using the smallest and the largest GO sheets, were incubated with 0.1 nM to 1 μM of MC and 1 ng/ml to 1 μg/ml of β-LG, respectively. The linear regression equations, the limit of detections (LOD) and limit of quantifications (LOQ) for each case are presented in the supporting information file. In fact, for both Apt/Cov and Imm/Cov sensors the LOD is lower for sensors made with small sizes of GO. For instance, Apt/Cov with 0.22 μm GO sheet sizes exhibits a LOD of 0.018 nM and LOQ of 0.062 nM compared with LOD = 0.25 nM and LQD = 0.820 nM for electrodes made with 300 μm GO sheet sizes. The same behaviour is observed for Imm/Cov (0.22 μm) sensors the LOD = 1.2 ng/ml and LOQ = 3.9 ng/ml compared to LOD = 2.60 ng/ml and LOQ = 8.70 ng/ml for Imm/Cov (300 μm). In parallel, for the Apt/Phys and Imm/Phys the lower LOD and LOQ are observed for electrodes made from large GO sheet sizes. For instance, Apt/Phys with 300 μm GO sheet sizes exhibits a LOD of 0.038 nM and LOQ of 0.129 nM compared with LOD of 0.088 nM and LQD of 0.295 nM for electrodes made with 0.22 μm GO sheet sizes. The same behaviour is observed for Imm/ Phys (300 μm) sensors, the LOD is 0.46 ng/ml and LOQ = 1.53 ng/ml compared to LOD = 0.79 ng/ml and LOQ = 2.65 ng/ml for Imm/Cov (300 μm). It thus appears that the choice of specific GO sheet size for a particular biosensing application should represent a compromise between sensitivity and reproducibility.

## Experimental section

### Materials and reagents

Anti-β-lactoglobulin antibody was obtained from Abcam (Cambridge, USA). Graphite powder (500 mesh), sulfuric acid (99.99%), phosphoric acid (85 wt. % in H_2_O), potassium permanganate, hydrogen peroxide solution 30% (w/w) in H_2_O, hydrochloric acid (37%), ethanol (> 99.8%), β-lactoglobulin (β-LG) (≥ 90%), potassium ferrocyanide (K_4_Fe(CN)_6_) (98.5–102.0%), potassium ferricyanide (K_3_Fe(CN)_6_) (≥ 99.0%), dipotassium hydrogen orthophosphate (≥ 99.0%), potassium dihydrogen orthophosphate (≥ 99.0%), sodium chloride (≥ 99.5%), sodium dodecyl sulfate (SDS) (≥ 99.0%), hydroxylamine hydrochloride (≥ 99.0%), 2-(N-morpholino) ethanesulfonic acid (MES) (≥ 99.5%), ovalbumin from chicken egg white (≥ 98%), Bovine serum albumin was purchased from Sigma (Ontario, Canada). EDC and N-NHS were obtained from Fisher Scientific (Ontario, Canada). A phosphate buffered saline PBS solution (10 mM, pH 7.4) was used for the preparation of the antibody and the β-lactoglobulin standard stock solutions and dilutions. Microcystin-LR (MC-LR), Microcystin-LA (MC-LA) (≥ 95% (HPLC)) and okadaic acid sodium salt (OA) (≥ 98% (HPLC)) were purchased from Enzo Life Sciences (Ontario, Canada). Standard MC-LR solutions were prepared by dissolving the toxin in binding buffer (50 mM Tris, pH 7.5, 150 mM NaCl, 2 mM MgCl_2_). Okadaic acid was firstly dissolved in methanol (0.1 g L^−1^) and subsequently diluted in the binding buffer. Unmodified Single-stranded DNA (ssDNA) aptamer for MC-LR (MCAP): 5′-GGC GCC AAA CAG GAC CAC CAT GAC AAT TAC CCA TAC CAC CTC ATT ATG CCC CAT CTC CGC-3′^48^ and NH_2_-modified aptamer (NH_2_-MCAP): 5′-NH_2_ (CH_2_)_6_/GGC GCC AAA CAG GAC CAC CAT GAC AAT TAC CCA TAC CAC CTC ATT ATG CCC CAT CTC CGC/-3′were synthesized by Integrated DNA Technologies (IDT Inc.) (Coralville, USA). Milli-Q water was used in the preparation of all solutions. Polycarbonate membrane filters were obtained from Sterlitech Corporation (WA, USA). Cellulose acetate and Nylon filters were obtained from Millipore Corporation (MA, USA). Disposable electrical printed (DEP) electrodes (three-electrode configuration, comprising a carbon working electrode, carbon counter and silver/silver chloride reference electrode) were obtained from BioDevice Technology (Nomi, Japan).

### Equipment

A centrifuge (Beckman Avanti J-25, Canada) was used for GO sheets preparation/separation protocols. Ultrasonication of GO dispersion was performed in a 50 W ultrasonicator (Shenzhen Co., China), at a frequency of 42 kHz. Ultrasonication temperature was controlled and always maintained at 55 °C. Scanning electron microscopy (SEM) images were acquired on a JEOL JSM7600F system operating at an accelerating voltage of 5 kV. Atomic force microscopy images, used to determine GO sheet size and thickness, were obtained using Veeco/Bruker AFM instrument in ScanAsyst mode. X-ray photoelectron spectroscopy (XPS) measurements were performed with XPS PHI 5,600-ci instrument (Physical Electronic, Inc., USA) using a Mg polychromatic source (MgKα = 1,253.6 eV) at 150 W. Raman spectroscopy measurements were performed using a micro-Raman spectrometer (RenishawInVia Reflex, Model: RM3000) with excitation from an argon ion laser beam (514 nm) in a backscattering geometry. ^13^C-SS NMR (MAS) spectra were recorded using a direct pulse sequence with broadband proton decoupling using a 600 MHz Varian Inova Unity spectrometer (Agilent, Santa Clara, CA) operating at frequencies of 150.874 MHz for ^13^C and 599.84 MHz for ^1^H. Magic angle spinning (MAS) was performed at a spinning frequency of 12.5 kHz. Data were analyzed using the Mestrenova® software (Mestrelab Research). An Autolab PGSTAT302N potentiostat (Eco Chemie, The Netherlands) controlled by NOVA software version 1.9 was used to perform all electrochemical experiments which were carried out at room temperature. The square wave voltammetry (SWV) was performed in 1 mM ferro/ferricyanide solution in PBS pH 7.4, parameters were^43^: amplitude = 20 mV, interval time = 0.04 s, step potential = -5 mV, scan rate = 125 mV s^−1^ and frequency = 25 Hz.

## Procedures

### Synthesis of graphene oxide

Graphene oxide was prepared using modified Hummers method^38^. In a typical experiment, 3 g of graphite powder, 360 mL of H_2_SO_4_ and 40 mL of H_3_PO_4_ were mixed in a 1L flask and stirred for 30 min at 55 °C. Then, 18 g of KMnO_4_ was added to the mixture in small portions to prevent the rapid temperature rise. The mixture was stirred continuously for 4.5 h and the temperature was kept at 55 °C. Several ultrasonication periods of 30 min were applied during the stirring time to exfoliate graphite oxide into graphene oxide (GO) sheets. The suspension was further treated by adding it to a mixture of H_2_O_2_ (10 mL, 30%) and water (600 mL) at 0 °C and stirred. This step is used to convert the residual permanganate and MnO_2_ into soluble MnSO_4_. The resulting suspension which has a bright yellow color is cleaned using a mixture of water, HCl and ethanol. The mixture is then washed with ethyl ether, filtered using Teflon filters and dried at 30 °C under vacuum. A suspension of GO was prepared by stirring the solid GO powder in water for 24 h.

### Separation of graphene oxide flakes

Separation of GO sheets according to their lateral size was realized by a size fractionation process involving a repeated ultracentrifugation step for 10 min at 4,000 rpm followed by separation of fractions and successive filtration using membranes with different pore sizes to obtain the required size range. Different centrifugation speeds and times were used to optimize the separation. The separated fractions were then dried and 1 mg/mL GO of each size was redispersed in water by shaking for 24 h to achieve maximum dispersion of the material. All GO dispersions with different sizes were stable in aqueous media for 1 year.

### Preparation of graphene oxide modified electrodes

The GO modified DEP electrodes were prepared by drop casting. 5 μL of GO solution (1 mg/mL in milli-Q water) was deposited onto the electrode surface and allowed to dry at room temperature. The excess of material was then removed from the electrode surface by gentle rinsing with milli-Q water. As illustrated in Figure S5 a sharp reduction peak at − 0.87 V is seen during the first cycle, similar to what was previously reported for the reduction of GO sheets on glassy carbon electrode surface^48^. Figure S 5B (supporting information) reports the evolution of the CVs recorded in the presence of [Fe (CN)_6_]^3−/4−^ probe for the bare SPCE electrode, GO and ERGO modified surfaces. The dependence of peak-to-peak separation (∆E_p_) and peak current (i_p_) values on the GO/ERGO sheet sizes are shown in Figure S 5C, 5D (supporting information). As observed, the ∆E_p_ decreases and the i_p_ increases following the reduction process because of the lower number of negatively charged groups and the higher conductivity of ERGO with respect to pristine GO. Finally, (Figure S5C,5D) also show that the GO materials with the large sheet size (> 300 μm) were the ones which showed the highest degree of reduction, followed by the small sizes then medium sizes.

### Characterization

For the AFM and SEM observation, freshly cleved mica substrates were soaked in an aqueous solution of 3-aminopropyltriethoxysilane (APTES; 12 μL of APTES in 20 mL of H_2_O) for 15 min. After being thoroughly rinsed with deionized H_2_O and blow-dried with nitrogen, the substrate was soaked in a solution of GO. Then, the substrate was taken out from the solution and left to air dry at room temperature for 30 min. For Raman spectroscopy analysis, the graphene oxide solution (1 mg/mL) was drop casted on a clean SiO_2_/Si substrate and left to air dry at room temperature. The XPS analyses were made on the GO modified electrodes.

### Physical immobilization of MC-LR Aptamer and β-LG antibody

5 μL of MC-LR aptamer in binding buffer or 5 μL of β-LG antibody in PBS buffer pH 7.4 at the optimum concentrations of 10 μM and 10 μg/ml, respectively, were incubated on the GO electrodes surface for 1 h until drying at room temperature. The electrodes were then washed with a buffer to remove the excess of non-adsorbed molecules.

### Covalent Immobilization of NH_2_-modified MC-LR aptamer and β-LG antibody

5 μL of 100 mM EDC+ 20 mM NHS in MES buffer solution pH 5.2 were deposited on the GO-modified electrode surfaces and left for 1 h in order to activate the carboxylic acid groups. Then, the electrodes were rinsed with PBS buffer solution and incubated with NH_2_-MCAP or β-LG antibody at the optimum concentrations of 10 μM and 10 μg/ml, respectively in PBS buffer for 3 h under a wet environment. Subsequently, the aptasensor was washed with PBS buffer containing 0.05% SDS and the immunosensor was washed with PBS+ 0.1% Tween 20 to remove non-specifically adsorbed NH_2_-MCAP and β-LG antibody, respectively. Then the electrodes were incubated with 0.1 M hydroxylamine hydrochloride for 30 min to deactivate any remaining carboxylic acid groups before another washing step with PBS buffer solution.

### Detection and selectivity experiments for B-lactoglobulin and microcystin-LR

The desired concentrations of MC-LR in binding buffer and β-LG in PBS buffer were incubated for 1 h on the surface of GO aptasensor and immunosensor, respectively. The electrodes were subsequently washed with binding buffer or PBS buffer with 0.1% Tween 20 and subjected to electrochemical measurements. Negative control and cross reactivity experiments were performed by incubating the aptasensors with MC-LA, OA in binding buffer and the immunosensors with OVA, BSA in PBS.

## Conclusions

Graphene oxide suspensions with different sheet sizes have been successfully separated. The morphology and structural properties of these samples were characterized and compared using AFM, SEM, XPS, Raman spectroscopy and NMR. The smallest graphene oxide sheets showed higher defect density and degree of oxidation based on the Raman spectroscopy and XPS results. The biosensing performance of the different GO samples was compared using DNA aptamer against microcystin-LR toxin as well as an antibody against β-lactoglobulin in label-free detection format. We observed different trends between the size of GO sheets and the performance of MC-LR aptasensors and β-LG immunosensors fabricated either using covalent attachment or physical adsorption. For the Apt/Phys, with increasing the size of the GO sheet, we observed a gradual increase in the aptasensor response. However, for the Apt/Cov, the smallest sized-GO sheets showed the highest signals. On the other hand, for the Imm/Phys and Imm/Cov, the optimal signals were obtained at intermediate GO sheet sizes. Moreover, good selectivity for the aptasensors prepared by either physical adsorption or covalent immobilization was observed. However, the immunosensors fabricated on the larger sized GO sheets does not display satisfactory selectivity towards β-LG compared to the smaller sheets. Good reproducibility was also observed for the small-sized GO sheets unlike the poor reproducibility of the sensors of large GO sheets. We found that the graphene-based electrodes should take in consideration amounts of edge and basal planes defects derived from oxygen containing functional groups. However, we found difficult to control the amount of defects which could be related to the GO (graphene particle) agglomeration or simply the folding of the aptamer or the protein occurring upon binding. Our results have shown strong dependency of the biosensors response signals, selectivity and reproducibility on the GO sheet size.

## Supplementary information

Supplementary information.
