# Supplementary material for: Probing the influence of graphene oxide sheets size on the performance of label-free electrochemical biosensors
Source: Sci Rep. 2020 Aug 12;10:13612. doi: 10.1038/s41598-020-70384-5 (PMC7424566; doi:10.1038/s41598-020-70384-5)
Supplement: Supplementary file 1 — Supplementary information. [file 41598_2020_70384_MOESM1_ESM.pdf]

<u>Supporting information</u>

Probing the Influence of Graphene Oxide Sheets Size on the Performance of Label-Free Electrochemical Biosensors

Shimaa Eissa[a,b,c], Jeanne N'diaye[a], Patrick Brisebois[a], Ricardo Izquierdo[a], Ana C. Tavares[b], Mohamed Siaj*[a]

[a]Dept. de Chimie et Biochimie, NanoQAM, CQMF, Université du Québec à Montréal, Montréal,

[b]Institut National de la Recherche Scientifique – Énergie, Matériaux et Télécommunications, 1650, Boul. Lionel-Boulet, Varennes, Québec, Canada, J3X 1S2

Québec, Canada, H3C 3P8

[c] Present address: Department of Chemistry, Alfaisal University, Al Zahrawi Street, Al Maather, Al Takhassusi Road, Riyadh 11533, Saudi Arabia

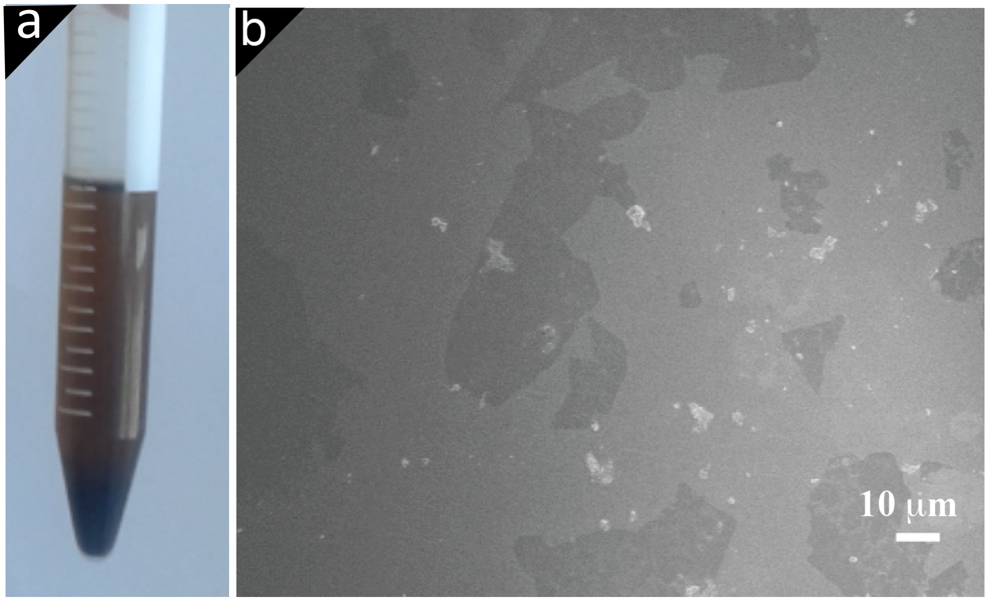

Figure S1 (a) Digital camera images of an ultracentrifuge tube after centrifugation, (b) SEM image of graphene oxide sheets separated only by centrifugation.

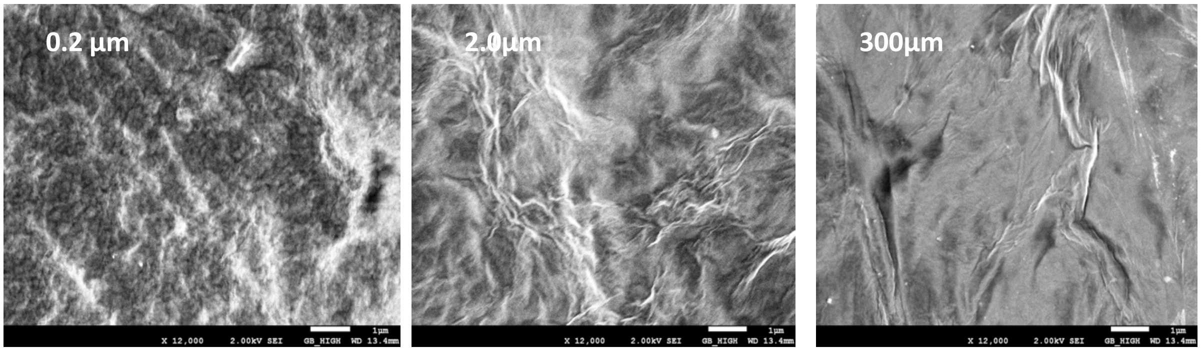

Figure S2 Scanning electron micrographs of the GO-modified electrodes (sheet sizes: 0.2, 0.7, >100 µm)

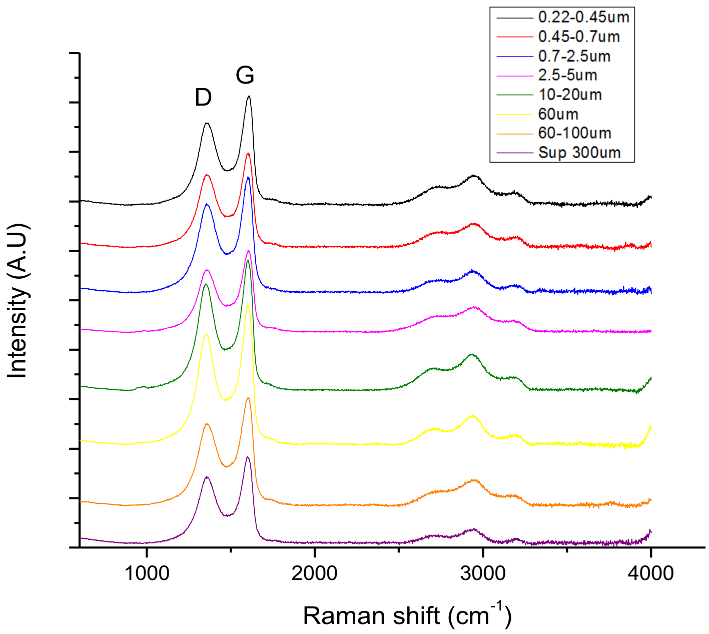

Figure S3 Raman spectra of the different GO samples

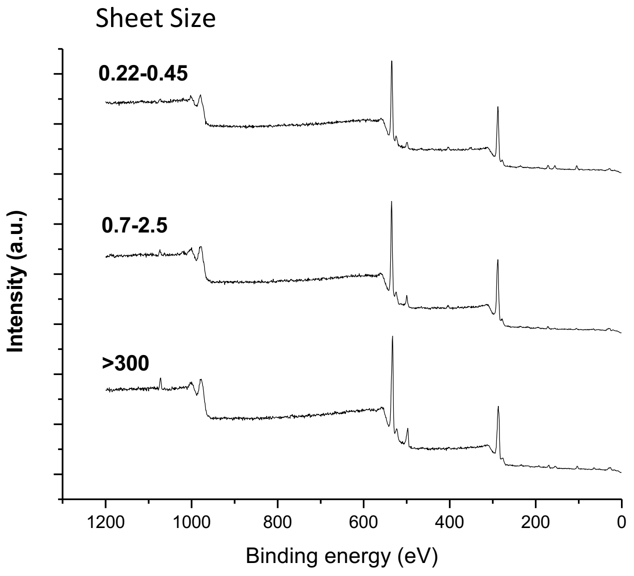

Figure S4 representative XPS survey spectrum for a graphene oxide sample exhibiting two predominant peaks that corresponds to core C1s at 286 eV and O1s at 534 eV. The C/O ratio for all GO sample, calculated from the atomic percentages, reveals that the C/O ratio slightly varies with the sheet size. The smallest size GO samples (0.22 and 0.45 μm) presented the lowest C/O ratio of 1.81, followed by 0.7 μm GO with a C/O ratio of 1.87, then 10, 60 μm GO with a C/O ratio of 2.06, and 300 μm GO with the highest C/O ratio of 2.12. These results indicate larger amount of oxygen-containing groups in the GO samples with the smaller sheets likely due to the higher amount of edges.

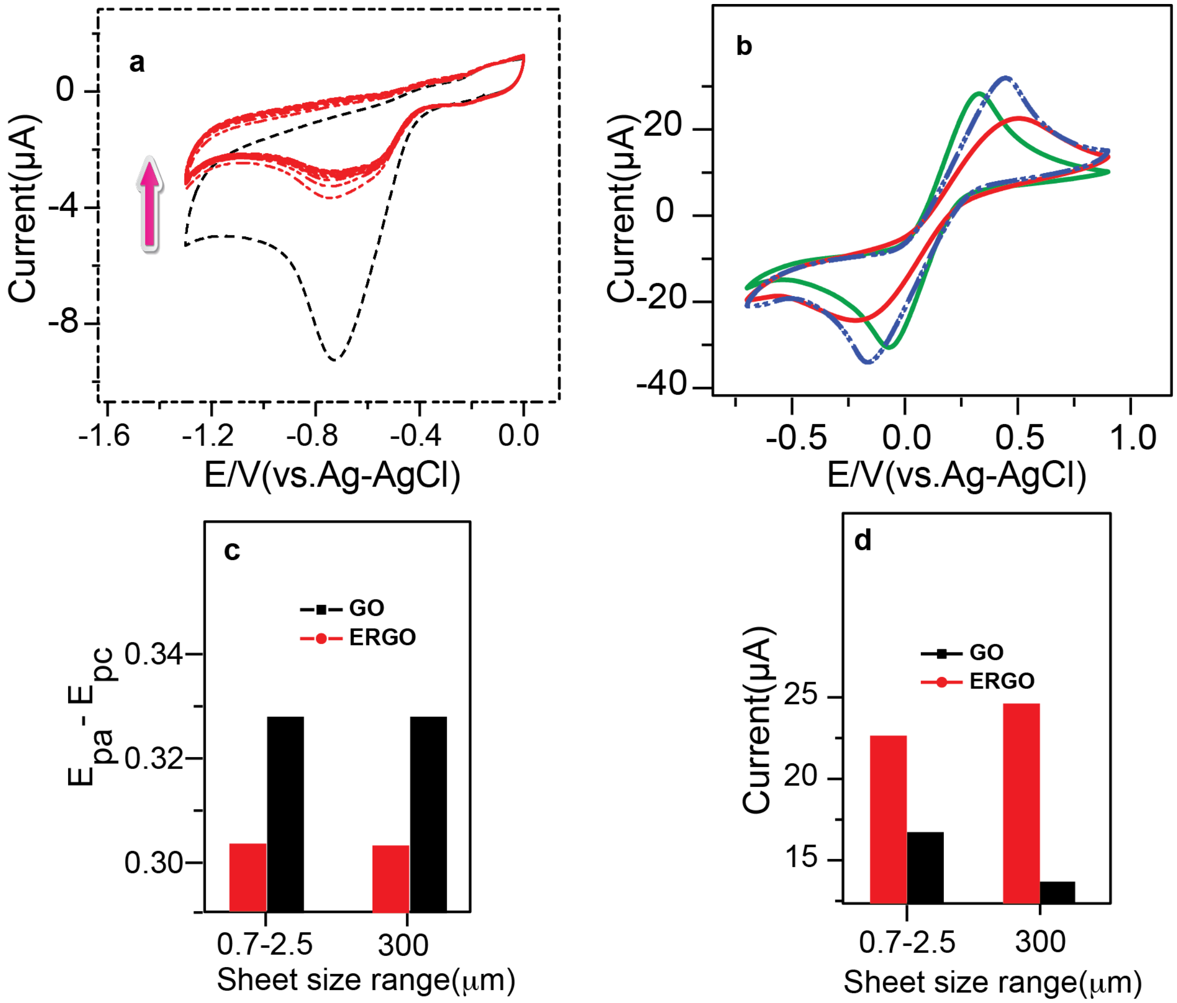

Figure S5. (A) Electrochemical reduction of GO/AP/SPCE surface (in 0.5 M NaCl) at a scan rate of 50 mV s^{-1}. (B) Cyclic voltammograms (CVs) of 5 mM $[Fe(CN)_6]^{3-/4-}$ probe in PBS, pH 7.4, for bare SPCE electrode (black), SPCE/GO (red), SPCE/ERGO (blue). The CVs were performed at a scan rate of 100mV/s. (C), (D) The effect of GO/ERGO sheet sizes on the peak to peak separation (ΔE_p) and anodic peak current (i_p) of 5 mM $[Fe(CN)_6]^{3-/4-}$ redox couple in PBS, pH 7.4.

As illustrated in Figure S5 a sharp reduction peak at −0.87 V is seen during the first cycle, similar to what was previously reported for the reduction of GO sheets on glassy carbon electrode surface [H. Wang, Q. Hao, X. Yang, L. Lu, X. Wang, ACS Applied Materials & Interfaces 2010, 2, 821-828.]. Figure S 5B (supporting information) reports the evolution of the CVs recorded in the

presence of $[Fe(CN)_6]^{3-/4-}$ probe for the bare SPCE electrode, GO and ERGO modified surfaces. The dependence of peak-to-peak separation (ΔE_p) and peak current (i_p) values on the GO/ERGO sheet sizes are shown in Figure S 5C and 5D (supporting information). As observed, the ΔE_p decreases and the i_p increases following the reduction process because of the lower number of negatively charged groups and the higher conductivity of ERGO with respect to pristine GO. Finally, (Figure S5C and 5D) also show that the GO materials with the large sheet size (> 300 µm) were the ones which showed the highest degree of reduction, followed by the small sizes then medium sizes.

LOD and LOQ for MC-LR (microcystin-LR)

The LOD is calculated as 3S/b where S is the standard deviation of the blank/background signal (the sensor incubated in buffer solutions)

	nM	nM
MC-LR/Apt-Phys	LOD	LOQ
300 µm	0.038	0.129
0,22 µm	0.088	0.295

MC-LR/Apt-Cov	LOD	LOQ
300 µm	0.250	0.820
0.22 µm	0.018	0.062

Apt/Phys-300 µm: A good linear relationship in the range of 0.1 nM to 1.0 µM was obtained between the MC-LR concentration and the analytical response (ip − i)/i% in binding buffer, which can be represented by the equation, (ip − i)/i% = 18.64 + 6.148 x C [nM], $R^2 = 0.9992$, with a detection limit (LOD) of **0.038 nM** (S/N = 3) and **LOQ = 0.129 nM.**

Apt/Phys-0.22 µm: A good linear relationship in the range of 0.1 nM to 1.0 µM was obtained between the MC-LR concentration and the analytical response (ip − i)/i% in binding buffer, which can be represented by the equation, (ip − i)/i% = 12.52 + 11.81 x C[nM], $R^2 = 0.9855$ with a detection limit (LOD) of **0.088 nM** (S/N = 3) and **LOQ = 0.295 nM.**

Apt/Cov-300 µm: A good linear relationship in the range of 1 nM to 1.0 µM was obtained between the MC-LR concentration and the analytical response (ip − i)/i% in binding buff er, which can be represented by the equation, (ip − i)/i% = 3,36 x C[nM] + 7.98; R^2 = 0.997, with a detection limit (LOD) of **0.25 nM** (S/N = 3) and **LOQ = 0.82 nM**.

Apt/Cov -0.22 µm: A good linear relationship in the range of 1 nM to 1.0 µM was obtained between the MC-LR concentration and the analytical response (ip − i)/i% in binding buffer, which can be represented by the equation, (ip − i)/i% = 9.81 x C[nM] + 10.22; R^2 = 0.9996 with a detection limit (LOD) of **0.018 nM** (S/N = 3) and **LOQ = 0.062 nM.**

LOD and LOQ for β-LG

ng/ml

beta-LG/Apt-Phys	LOD	LOQ
300 µm	0.46	1.53
0.22 µm	0. 79	2.65

ng/ml

beta-LG/Apt-Cov	LOD	LOQ
300 µm	2.60	8.70
0.22 µm	1.20	3.90

Imm/Phys-300 µm: A good linear relationship in the range of 0.001 µg/ml to 1.0 µg/ml was obtained between the B-LG concentration and the analytical response (ip − i)/i% in binding buffer, which can be represented by the equation, ((ip − i)/i% = 4,92 x C [ng/ml] + 14,93; R^2 = 0,9993, with a detection limit (LOD) of **0.46 ng/ml** (S/N = 3) and **LOQ = 1.53 ng/ml.**

Imm/Phys-0.22 µm: A good linear relationship in the range of 0.001 µg/ml to 1.0 µg/ml was obtained between the B-LG concentration and the analytical response (ip − i)/i% in binding buffer, which can be represented by the equation, (ip − i)/i% = 13,41 x C [ng/ml] + 11,87; R^2 = 0,9848, with a detection limit (LOD) of **0.79 ng/ml** (S/N = 3) and **LOQ = 2.65 ng/ml.**

Imm/Cov-300 µm:A good linear relationship in the range of 0.01 µg/ml to 1.0 µg/ml was obtained between the B-LG concentration and the analytical response (ip − i)/i% in binding buffer, which can be represented by the equation, (ip − i)/i% = 1.91 x C [ng/ml] + 2.32; R^2 = 0.984, with a detection limit (LOD) of **2.60 ng/ml** (S/N = 3) and **LOQ = 8.70 ng/ml**.

<u>Imm/Cov -0.22 µm:</u> A good linear relationship in the range of 0.01 µg/ml to 1.0 µg/ml was obtained between the B-LG concentration and the analytical response (ip − i)/i% in binding buffer, which can be represented by the equation, (ip − i)/i% = 9.74 x C [ng/ml] + 10.49; R^2 = 0.9997 with a detection limit (LOD) of **1.2 ng/ml** (S/N = 3) and **LOQ = 3.9 ng/ml.**
